# New energy power system operation security evaluation based on the SWOT analysis

**DOI:** 10.1038/s41598-022-16444-4

**Published:** 2022-07-25

**Authors:** Saniye Maihemuti, Weiqing Wang, Jiahui Wu, Haiyun Wang

**Affiliations:** College of Electrical Engineering, Xinjiang University, Urumqi, 830047 CO People’s Republic of China

**Keywords:** Electrical and electronic engineering, Energy science and technology

## Abstract

Grid-connection of new energy is highly important in promoting the use of clean and renewable energy. However, it will bring huge risks to the power grid operation security, such as frequency stability, voltage stability, small signal stability, and transient stability, etc.,. In the study, SWOT (Strengths, Weaknesses, Opportunities, and Threats) analysis has been employed to construct 24 kinds of internal and external evaluation factors and 8 kinds of improvement strategies, for assessing operation security prospective with new energy power system of HM in China. The weights of SWOT factors are determined with the fuzzy-AHP method. Moreover, the fuzzy-MARCOS approach is used to select the most suitable strategies for power system operation security effective implementation. The reported research reveals that new energy in HM area not only has an ample potential for full development and generating electricity, but also brings operation security problems due to large-scale grid connection. Therefore, 8 kinds of improvement strategies are suggested to encourage the government to exploit and develop new resources, improve the investment pay, power generation and transmission technologies to mitigate the current energy crisis, and increase the energy security for sustainable development of the country. The methodology proposed herein is applicable with a case study concerning the operation security prospective of HM power grid, and all phases of the comparative analysis and sensitivity analysis illustrate the validity of MARCOS method. Furthermore, the ranked order of strategies is obtained as *A*_2_ > *A*_6_ > *A*_5_ > *A*_1_ > *A*_8_ > *A*_7_ > *A*_4_ > *A*_3_. The three most important strategies are *A*_2_, *A*_6_ and *A*_5_, i.e., “improving the technical establishment to encourage efficient and cheap electricity production”, “strive to build local permanent load, and reduce the risk of long-distance and high-capacity transmission”, “taking advantage of government incentives and investment to modify the irrational energy policies and energy planning”, respectively.

## Introduction

HM is one of the rich regions in terms of new energy resources, which are wind and solar resources, and coal resources. Relying on the perfect Northwest China power transmission channel, HM has accelerated the construction of 10 million kW coal power base, 10 million kW wind power base and 1 million kW solar thermal power generation demonstration base; and has built the largest wind and fire binding transmission HVDC base in China. The development of wind power technology in HM has reached 75.498 million kW, accounting for 62.9% of the total technology development in Northwest China. In terms of solar energy resources, HM has 3170–3380 h of sunshine throughout the year, and is one of the areas with most abundant sunshine hours in China. Although HM power grid offers such great advantages in the development of new energy, the large-scale grid connection and long-distance transmission of new energy raises concerns about the operation security of HM power grid. Therefore, the security and stability of HM power grid has become the focus of our research.

Power system security is a characteristic of power system in the operation process, which reflects the ability of the system to continue supplying power to users with required parameters after experiencing possible disturbances^1,2^. Although most countries take “safety first” as a policy to guide the operation of power system, some countries even raise the power system to the national strategic defense system^3,4^, even so, blackouts still occur from time to time, indicating that the ability of current power systems is still unsatisfactory to deal with the emergencies^5,6^.

At present, with the continuous development of China’s power system and interconnection scale, China’s power workers have built a strong and stable protection and control system based on three lines of defense to ensure a stable operation, which ensures the stability of the power grid and the power quality supplied to users, to a great extent. Tant is so say, power outage accidents in large areas are almost impossible to occur in China. The first line of defense relies on the reliable and rapid action of relay protection elements, to ensure that the system can restore stable operation in time and supply power normally in case of a single fault. Progressively, the second line of defense includes a series of emergency control measures, which can ensure a stable operation of the system at a low cost, after suffering from low probability but serious disturbance. The main measures include machine cutting, load cutting, local splitting and DC modulation. In engineering practice, the control framework of “off-line pre-decision and real-time matching” is often used to form a large number of emergency control strategy tables of the expected faults in the off-line stage. The significance of the third line of defense is that when the system encounters some rare serious faults and can no longer maintain a stable operation, it must prevent the system collapse and minimize the load loss, that is, reasonable disconnection. However, there is no mature scientific connection between the three lines of defense at present. These lines of defense are still event driven and act gradually according to the development of events.

With the rapid development of national economy and the increasing improvement of people’s living standards, people’s demand and dependence on power are becoming greater and greater, and consequently, the requirements for power supply reliability are becoming stronger and stronger^7,8^. Certainly, due to the impact of natural environment, new energy power generation has some randomness and intermittency. For example, when wind power is connected to the power grid, the security and stability of the power grid will be affected by the wind power characteristics, wind power installed capacity, system scale of wind power connected to the power grid, power structure and layout, and load characteristics, etc. Moreover, the installed capacity of new energy affects the frequency stability of the system, thereby affecting the power quality of power grid and the normal operation of some frequency sensitive loads. In addition, new energy has weak inertia support, strong output uncertainty, poor frequency regulation ability and damping characteristics, resulting in the increasingly prominent problem related to system frequency stability. When the new energy loses its output due to energy shutdown or stall, the frequency of the power system will be reduced, especially when the penetration level of new energy is high, which will directly affect the frequency stability of the system. Hence, the new energy itself cannot provide reactive power compensation to the system, thus it worsens the voltage level, reduces the power quality of the system, and affects the voltage stability of the system. In addition, new energy is a source of interference for the power system, however, when the system fails, and the fault is not removed in time, transient voltage instability will occur. All these issues demand a higher amount of technical attention for the safe and stable operation of new energy power system^9–11^. Modern power system has gradually developed into a complex large-scale system with the characteristics of multi-level structure, multi time scale, multiple control parameters, dynamics, real-time, nonlinearity, openness, wide area, uncertainty, non-autonomy and social economy^12–14^. Therefore, the security level of a power system is difficult to be characterized using a single index. However, the conventional security analyses, whether static security analysis^8,15–17^ or dynamic security analysis^18–22^, usually can only analyze one aspect of the power system security, which is certainly not enough to describe the power system security as a whole. Therefore, only through comprehensive, multi-level and multi angle analysis of all the aspects of the system including the randomness and fuzziness of components, loads and external conditions, with the help of comprehensive evaluation method, can we obtain an objective and overall understanding of the power system security^23^.

The so-called comprehensive evaluation refers to the objective, fair and reasonable evaluation of different aspects of the evaluated object. Its core includes the index system of the evaluated object and the selected evaluation method^24^. At present, the Multi-Criteria Decision-Making (MCDM) method corresponds to a powerful tool being widely used for comprehensive evaluation, such as analytic hierarchy process (AHP)^23–27^, entropy weight (EW)^25–28^, cloud method (CM)^24,28^, grey relation analysis (GRA)^29^, analytic network process (ANP)^30–32^, Decision Making Trialand Evaluation Laboratory (DEMETEL)^31–35^, Više-Kriterijumska Optimizacija I Kompromisno Rešenje (VIKOR)^33–39^, and technique for order of preference by similarity to ideal solution (TOPSIS)^28,36–39^ evaluation method based on evidence theory. These MCDM evaluation methods are currently being used for ranking of indicators, risk identification and evaluation, sustainable development evaluation between countries, economic evaluation, social evaluation, environmental protection evaluation, technical evaluation, and other issues. However, it has been hardly ever used in the power system security evaluation. In addition, the SWOT analysis with internal (**S**trengths and **W**eaknesses) and external factors (**O**pportunities and **T**hreats) is also a powerful tool, which helps to reveal different strategies for decision-makers and participants^40^. SWOT analysis is widely used in future prospective in different fields, but is not sufficient for decision making. Alternatively, MCDM methods can be helpful to overcome such problem^41–46^, which are used to make based on pair-wise comparison of both criteria and alternatives^43^. For this reason, one of the MCDM methods— AHP is used for SWOT analysis in this study. The SWOT-fuzzy AHP has been used to perform the strategic renewable energy resources selection for Pakistan^47^. Meanwhile, the integrated SWOT-AHP and Fuzzy-TOPSIS approach has been employed for evaluating the strategies for sustainable energy planning in Pakistan^48^. Nevertheless, there is no single study that utilizes SWOT-AHP approach to evaluate and select the optimal improvement strategy for power system operation security prospective.

In this study, the proposed SWOT-fuzzy AHP-MARCOS methodology is utilized for power system operation security perspective evaluation and investigating the strategic advantages in a case study of HM power grid in Northwest China. The obtained results are compared with VIKOR and TOPSIS methods to validate the proposed integrated SWOT-fuzzy AHP method and measurement of alternatives and ranking according to compromise solution (MARCOS) methodology^39^.

## Proposed method

This section first explains the SWOT method, its components, and its use in the literature. Following that, the proposed methodology is presented.

### SWOT analysis

**Figure 1:**
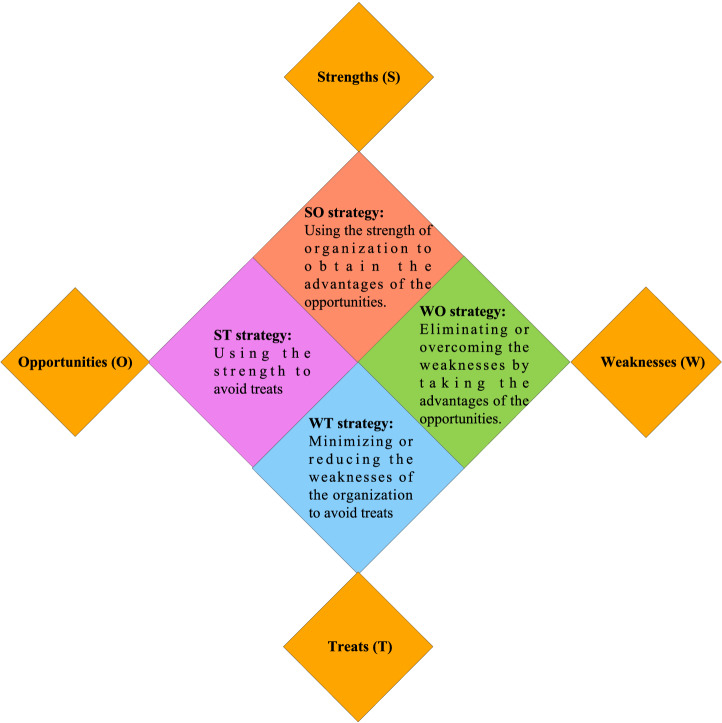
The SWOT strategies.

The SWOT analysis is a commonly used strategic analysis method that helps to identify the capabilities or deficiencies of an organization while considering the grid opportunities and threats for the future. However, SWOT analysis method can not quantitatively evaluate the factors, and can not objectively compare the priority between factors. Furthermore, a SWOT matrix may contain different strategies concerning its dimensions and factors, which are prepared based on strength-opportunity (SO), weakness-opportunity (WO), strength-threat (ST) and weakness-threat (WT) strategies; The strategies related to SWOT dimensions are given in Fig. 1.

Some of the advantages of SWOT analysis are listed as follows:

- It allows attention to the positive and negative aspects of the external and internal environment of the system;
- By understanding the weaknesses, it helps to identify opportunities to take advantage of strengths and eliminate threats;
- Determining the SWOT factors of the research object through group discussions provides the basis and support for the strategic decision-making;
- It can be used at different levels in different industries, organizations, countries or governments, such as individual, national, organizational, and international levels.

The combination of SWOT analysis and MCDM method, such as AHP, has been used to determine the weight of each SWOT factor. Reference^40^ reveals that most of the studies use the AHP method for prioritizing the SWOT factors and determining their weights, and recent studies employ the fuzzy AHP method combined with SWOT analysis and other MCDM method, such as TOPSIS, MABAC, DEMATEL, VIKOR and MARCOS. The use of AHP method for calculating the weights of SWOT factors and the need for a selection method such as MARCOS——an integrated SWOT-based fuzzy AHP-MARCOS method illustrates and their advantages are introduced next.

### The integrated SWOT-fuzzy AHP-MARCOS method

Various MCDM methods developed for decision-making processes in the literature have been utilized to solve different problems concerning the decision-making in various areas. Moreover, MCDM methods are also widely used in the studies focusing on power system security assessment.

#### The AHP method

AHP is a popular MCDM technology in comprehensive evaluation research proposed by American operations research scientists in 1970s^24^. It is one of the most suitable methods for natural resource planning and environmental assessment. This method requires less quantitative data and information, and is simple and practical. The SWOT analysis method needs to be quantified by incorporating the SWOT model into a hierarchical structure through AHP method, and therefore, it is completely reasonable to use AHP method in this study.

#### The MARCOS method

MARCOS is one of the novel MCDM methods proposed by Stevi´c et al., which is designed to evaluate alternatives concerning multiple decision factors based on ideal and anti-ideal solutions and provides the determination of utility degrees for both of these solutions. Recently, References^48^ utilized the MARCOS method, and integrated it with the AHP method. Owing to its flexibility about the analysis of the expert preferences without considering the type of scale, it maintains the stability even if the range of standards and alternatives is wide. They validated the advantages of MARCOS methodology compared to traditional multi-criteria techniques including: Multi-Attributive Border Approximation area Comparison (MABAC)^26^, Additive Ratio Assessment (ARAS)^27^, Weighted Aggregated Sum Product Assessment (WASPAS)^28^ and TOPSIS^29^. MARCOS has been successfully applied in different areas by considering the above-mentioned examples from the literature. However, the use of MARCOS method for the strategy selection emphasizes the contributions and originality of this study. Compared to other selection methods such as TOPSIS, MABAC, COPRAS and VIKOR, the proposed combined method generates quite consistent results. Moreover, it provides a compromise solution concerning ideal and anti-ideal solutions, thereby ensuring a satisfactory performance in a fuzzy environment.

#### The integrated methodology

**Figure 2:**
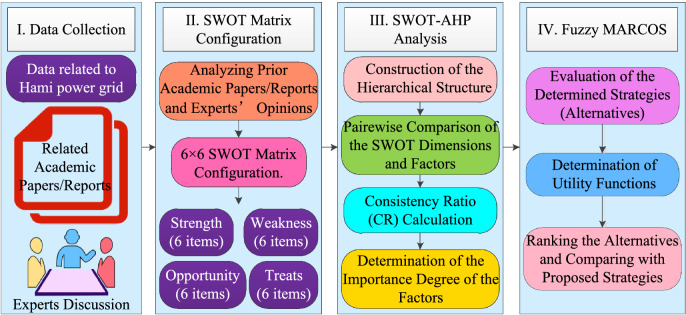
The framework of the proposed methodology.

In the proposed method, AHP is deployed to obtain the criteria weights with SWOT, and MARCOS is utilized to evaluate the alternatives and select the best alternative to benefit from their strengths. The general framework and the flow of proposed integrated methodology are presented in Fig. 2.

## Proposed methodology

This section elaborates the computational steps of the integrated SWOT-fuzzy AHP-MARCOS methodology^39^.

**Step 1**: Construct the SWOT matrix and factors determined by the experts.

**Step 2**: Construct a hierarchical structure concerning the goal and SWOT factors.

**Step 3**: Obtain the judgments of the experts: determine the priorities of the criteria using the linguistic terms given in Table 1.

**Table 1:** TFN fuzzy scale.

Intensity of importance	Fuzzy number	Definition	Membership function
1	$\tilde{1}$	Poor significant (PS)	(1, 1, 2)
3	$\tilde{3}$	Moderate significant (MS)	(2, 3, 4)
5	$\tilde{5}$	Strong significant (SS)	(4, 5, 6)
7	$\tilde{7}$	Absolutely significant (AS)	(6, 7, 8)
9	$\tilde{9}$	Absolutely more significant (AMS)	(8, 9, 10)

A Fuzzy comparison matrix ${\tilde{\mathbf{A}}}$ is constructed at this step using the TFN as follows:

1 $$ {\tilde{\mathbf{A}}} = \left[ {\begin{array}{*{20}l} 1 \hfill & {\tilde{a}_{12} } \hfill & \cdots \hfill & {\tilde{a}_{1n} } \hfill \\ {\tilde{a}_{21} } \hfill & 1 \hfill & \cdots \hfill & {\tilde{a}_{2n} } \hfill \\ \vdots \hfill & \vdots \hfill & \ddots \hfill & \vdots \hfill \\ {\tilde{a}_{n1} } \hfill & {\tilde{a}_{n2} } \hfill & \cdots \hfill & 1 \hfill \\ \end{array} } \right] $$

where $\tilde{a}_{ij} = 1$, if *i* = *j* and $\tilde{a}_{ij} = \tilde{1},{\kern 1pt} {\kern 1pt} \tilde{3},{\kern 1pt} {\kern 1pt} {\kern 1pt} \tilde{5},{\kern 1pt} {\kern 1pt} {\kern 1pt} \tilde{7},{\kern 1pt} {\kern 1pt} {\kern 1pt} \tilde{9}$ or $\tilde{a}_{ij} = \tilde{1}^{ - 1} ,{\kern 1pt} {\kern 1pt} \tilde{3}^{ - 1} ,{\kern 1pt} {\kern 1pt} {\kern 1pt} \tilde{5}^{ - 1} ,{\kern 1pt} {\kern 1pt} {\kern 1pt} \tilde{7}^{ - 1} ,{\kern 1pt} {\kern 1pt} {\kern 1pt} \tilde{9}^{ - 1}$ where *i* ≠ *j*.

After constructing the fuzzy comparison matrix, AHP method and a MATLAB program are used to calculate the weights of fuzzy AHP.

**Step 4**: Get the judgments of the experts to evaluate the alternatives using Table 2, to obtain the group decision matrix ${\tilde{\mathbf{X}}}$, where ${\tilde{\mathbf{A}}}(AI)$ and ${\tilde{\mathbf{A}}}(AAI)$ are the ideal and anti-ideal solutions, respectively.

2 $$ \begin{gathered} ext.{\kern 1pt} {\kern 1pt} {\kern 1pt} {\kern 1pt} {\kern 1pt} {\kern 1pt} {\kern 1pt} {\kern 1pt} {\kern 1pt} {\kern 1pt} {\kern 1pt} {\kern 1pt} {\kern 1pt} {\kern 1pt} {\kern 1pt} {\kern 1pt} {\kern 1pt} {\kern 1pt} {\kern 1pt} {\kern 1pt} {\kern 1pt} {\kern 1pt} {\kern 1pt} {\kern 1pt} {\kern 1pt} {\kern 1pt} {\kern 1pt} {\kern 1pt} {\kern 1pt} {\kern 1pt} {\kern 1pt} {\kern 1pt} {\kern 1pt} {\kern 1pt} {\kern 1pt} {\kern 1pt} {\kern 1pt} {\kern 1pt} {\kern 1pt} \tilde{C}_{1} {\kern 1pt} {\kern 1pt} {\kern 1pt} {\kern 1pt} {\kern 1pt} {\kern 1pt} {\kern 1pt} {\kern 1pt} {\kern 1pt} {\kern 1pt} {\kern 1pt} {\kern 1pt} {\kern 1pt} {\kern 1pt} {\kern 1pt} {\kern 1pt} {\kern 1pt} {\kern 1pt} \tilde{C}_{2} {\kern 1pt} {\kern 1pt} {\kern 1pt} {\kern 1pt} {\kern 1pt} {\kern 1pt} {\kern 1pt} {\kern 1pt} {\kern 1pt} {\kern 1pt} {\kern 1pt} {\kern 1pt} {\kern 1pt} {\kern 1pt} {\kern 1pt} {\kern 1pt} {\kern 1pt} {\kern 1pt} \cdots {\kern 1pt} {\kern 1pt} {\kern 1pt} {\kern 1pt} {\kern 1pt} {\kern 1pt} {\kern 1pt} {\kern 1pt} {\kern 1pt} {\kern 1pt} {\kern 1pt} {\kern 1pt} {\kern 1pt} {\kern 1pt} {\kern 1pt} {\kern 1pt} \tilde{C}_{n} \hfill \\ {\tilde{\mathbf{X}}} = \begin{array}{*{20}l} {{\tilde{\mathbf{A}}}(AAI)} \hfill \\ {{\tilde{\mathbf{A}}}_{1} } \hfill \\ {{\tilde{\mathbf{A}}}_{2} } \hfill \\ \vdots \hfill \\ {{\tilde{\mathbf{A}}}_{m} } \hfill \\ {{\tilde{\mathbf{A}}}(AI)} \hfill \\ \end{array} \left[ {\begin{array}{*{20}c} {\tilde{X}_{ai1} } & {\tilde{X}_{ai2} } & \cdots & {\tilde{X}_{ain} } \\ {\tilde{X}_{11} } & {\tilde{X}_{12} } & \cdots & {\tilde{X}_{1n} } \\ {\tilde{X}_{21} } & {\tilde{X}_{22} } & \cdots & {\tilde{X}_{ai1} } \\ \vdots & \vdots & \ddots & \vdots \\ {\tilde{X}_{m1} } & {\tilde{X}_{m2} } & \cdots & {\tilde{X}_{mn} } \\ {\tilde{X}_{id1} } & {\tilde{X}_{id2} } & \cdots & {\tilde{X}_{idn} } \\ \end{array} } \right] \hfill \\ \end{gathered} $$

3 $$ {\tilde{\mathbf{A}}}(AAI) = \left\{ {\begin{array}{*{20}c} {\mathop {\min }\limits_{i} \tilde{x}_{ij} {\kern 1pt} {\kern 1pt} {\kern 1pt} {\kern 1pt} {\kern 1pt} {\kern 1pt} {\kern 1pt} {\kern 1pt} {\kern 1pt} if{\kern 1pt} {\kern 1pt} {\kern 1pt} {\kern 1pt} {\kern 1pt} j \in B{\kern 1pt} } \\ {\mathop {\max }\limits_{i} \tilde{x}_{ij} {\kern 1pt} {\kern 1pt} {\kern 1pt} {\kern 1pt} {\kern 1pt} {\kern 1pt} {\kern 1pt} {\kern 1pt} {\kern 1pt} {\kern 1pt} if{\kern 1pt} {\kern 1pt} {\kern 1pt} {\kern 1pt} {\kern 1pt} j \in C} \\ \end{array} } \right. $$

4 $$ {\tilde{\mathbf{A}}}(IA) = \left\{ {\begin{array}{*{20}c} {\mathop {\max }\limits_{i} \tilde{x}_{ij} {\kern 1pt} {\kern 1pt} {\kern 1pt} {\kern 1pt} {\kern 1pt} if{\kern 1pt} {\kern 1pt} {\kern 1pt} {\kern 1pt} {\kern 1pt} j \in B} \\ {\mathop {\min }\limits_{i} \tilde{x}_{ij} {\kern 1pt} {\kern 1pt} {\kern 1pt} {\kern 1pt} {\kern 1pt} if{\kern 1pt} {\kern 1pt} {\kern 1pt} {\kern 1pt} {\kern 1pt} j \in C} \\ \end{array} } \right. $$

**Table 2:** Evaluation scale for alternatives.

Linguistic term	Triangular fuzzy numbers
Rarely poor (RP)	(0.1, 0.1, 0.1)
Highly poor (HP)	(0.1, 0.1, 0.3)
Poor (P)	(0.1, 0.3, 0.3)
Middle poor (MP)	(0.3, 0.3, 0.5)
Middle (M)	(0.3, 0.5, 0.5)
Middle good (MG)	(0.5, 0.5, 0.7)
Good (G)	(0.5, 0.7, 0.7)
Highly good (HG)	(0.7, 0.7, 0.9)
Rarely good (RG)	(0.7, 0.9, 0.9)

**Step 5**: Normalize the ${\tilde{\mathbf{X}}}$ to obtain ${\tilde{\mathbf{N}}} = \left[ {\tilde{n}_{ij} } \right]_{m \times n}$ as follows:

5 $$ \tilde{n}_{ij} = \left( {n_{ij}^{l} ,n_{ij}^{m} ,n_{ij}^{u} } \right) = \frac{{\tilde{x}_{id} }}{{\tilde{x}_{ij} }} = \left( {\frac{{x_{id}^{l} }}{{x_{ij}^{u} }},\frac{{x_{id}^{l} }}{{x_{ij}^{m} }},\frac{{x_{id}^{l} }}{{x_{ij}^{l} }}} \right)if{\kern 1pt} {\kern 1pt} {\kern 1pt} {\kern 1pt} {\kern 1pt} j \in {\text{Cost}}{\kern 1pt} {\kern 1pt} {\kern 1pt} {\text{criteria}} $$

6 $$ \tilde{n}_{ij} = \left( {n_{ij}^{l} ,n_{ij}^{m} ,n_{ij}^{u} } \right) = \frac{{\tilde{x}_{ij} }}{{\tilde{x}_{id} }} = \left( {\frac{{x_{ij}^{l} }}{{x_{id}^{u} }},\frac{{x_{ij}^{m} }}{{x_{id}^{u} }},\frac{{x_{ij}^{u} }}{{x_{id}^{u} }}} \right)if{\kern 1pt} {\kern 1pt} {\kern 1pt} {\kern 1pt} {\kern 1pt} {\kern 1pt} j \in {\text{Benefit}}{\kern 1pt} {\kern 1pt} {\kern 1pt} {\text{criteria}} $$

**Step 6**: Obtain the weighted fuzzy matrix ${\tilde{\mathbf{V}}}$ where weight coefficients of the criterion $\tilde{\omega }_{j} = \left( {\omega_{j}^{l} ,\omega_{j}^{m} ,\omega_{j}^{u} } \right)$ are calculated using fuzzy-AHP method:

7 $$ {\tilde{\mathbf{V}}} = \left[ {\tilde{v}_{ij} } \right]_{m \times n} = \left( {v_{ij}^{l} ,v_{ij}^{m} ,v_{ij}^{u} } \right) = \tilde{n}_{ij} \otimes \tilde{\omega }_{j} $$

**Step 7**: Calculate the utility degree ${\tilde{\mathbf{K}}}_{i}$:

8 $$ \tilde{K}_{i}^{ - } = \frac{{\tilde{S}_{i} }}{{\tilde{S}_{ai} }} = \left( {\frac{{s_{i}^{l} }}{{s_{ai}^{u} }},\frac{{s_{i}^{m} }}{{s_{ai}^{m} }},\frac{{s_{i}^{u} }}{{s_{ai}^{l} }}} \right) $$

9 $$ \tilde{K}_{i}^{ + } = \frac{{\tilde{S}_{i} }}{{\tilde{S}_{id} }} = \left( {\frac{{s_{i}^{l} }}{{s_{id}^{u} }},\frac{{s_{i}^{m} }}{{s_{id}^{m} }},\frac{{s_{i}^{u} }}{{s_{id}^{l} }}} \right) $$

where $\tilde{S}_{i} = (s_{i}^{l} ,s_{i}^{m} ,s_{i}^{u} )$ indicates the addition of elements of ${\tilde{\mathbf{V}}}$:

10 $$ \tilde{S}_{i} = \sum\limits_{i = 1}^{n} {\tilde{v}_{ij} } $$

**Step 8**: Determine the utility functions for ideal $f(\tilde{K}_{i}^{ + } )$ and anti-ideal $f(\tilde{K}_{i}^{ - } )$ solutions as follows:

11 $$ f\left( {\tilde{K}_{i}^{ + } } \right) = \frac{{\tilde{K}_{i}^{ - } }}{{df_{crisp} }} = \left( {\frac{{k_{i}^{ - l} }}{{df_{crisp} }},\frac{{k_{i}^{ - m} }}{{df_{crisp} }},\frac{{k_{i}^{ - u} }}{{df_{crisp} }}} \right) $$

12 $$ f\left( {\tilde{K}_{i}^{ - } } \right) = \frac{{\tilde{K}_{i}^{ + } }}{{df_{crisp} }} = \left( {\frac{{k_{i}^{ + l} }}{{df_{crisp} }},\frac{{k_{i}^{ + m} }}{{df_{crisp} }},\frac{{k_{i}^{ + u} }}{{df_{crisp} }}} \right) $$

**Step 9**: Calculate the utility functions $f\left( {K_{i} } \right)$ to determine the rank of the alternatives:

13 $$ f\left( {K_{i} } \right) = \frac{{K_{i}^{ + } + K_{i}^{ - } }}{{1 + \frac{{1 - f(K_{i}^{ + } )}}{{f(K_{i}^{ + } )}} + \frac{{1 - f(K_{i}^{ - } )}}{{f(K_{i}^{ - } )}}}} $$

## Application of the proposed methodology

This section explains the HM power grid and its effect on the power system security. Following that, the conducted case study is illustrated.

### Introduction of HM power grid

In this section, the power system security is evaluated considering the actual specifications of HM power grid in China, which is a mainland city with a wealth of new energy resources. The HM power grid located in the hub of the “silk road” the ancient times. The unique natural environment makes the HM power grid extremely rich in wind and solar energy resources. In addition, it has a vast area and relatively flat terrain, where large scale wind farms and photovoltaic farms are distributed, and the distribution scale is gradually expanding. Moreover, HM is the first cross-regional UHV transmission channel to absorb wind power and other new energy resources in Northwest China. Also, it facilitates the first new energy and thermal power bundling through UHVDC power transmission project in China^49^.

**Figure 3:**
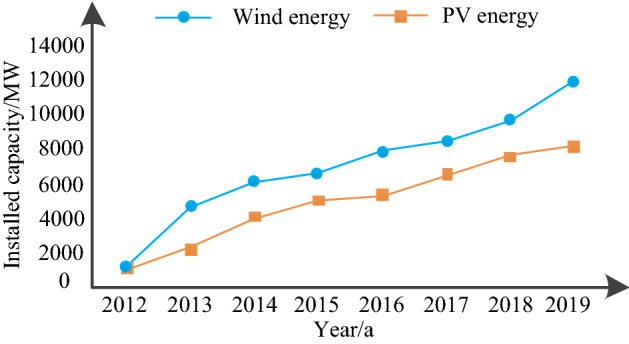
Growth rate of wind energy and solar energy scale in HM region.

According to the 13th five-year plan of HM Region, it was expected that the installed capacity of wind power would reach 12,100 MW and that of photovoltaic power would reach 9000 MW by 2020, of which the growth rate from 2012 to 2019 would increase year by year. Figure 3 shows the growth rate curve of wind and photovoltaic power installed capacity at HM power grid. The wind power scale and photovoltaic installed capacity of HM region continued to grow rapidly from 2012 to 2019, where the growth rate of wind power installed capacity was greater than that of the photovoltaic power. It can be seen that HM region, as the main gathering place of wind energy, is the key to new energy power generation in the whole China. As a hub connected with the mainland power grid, the energy security of HM region evidently affects the energy security of the whole China power system transmission.

### SWOT factors

**Figure 4:**
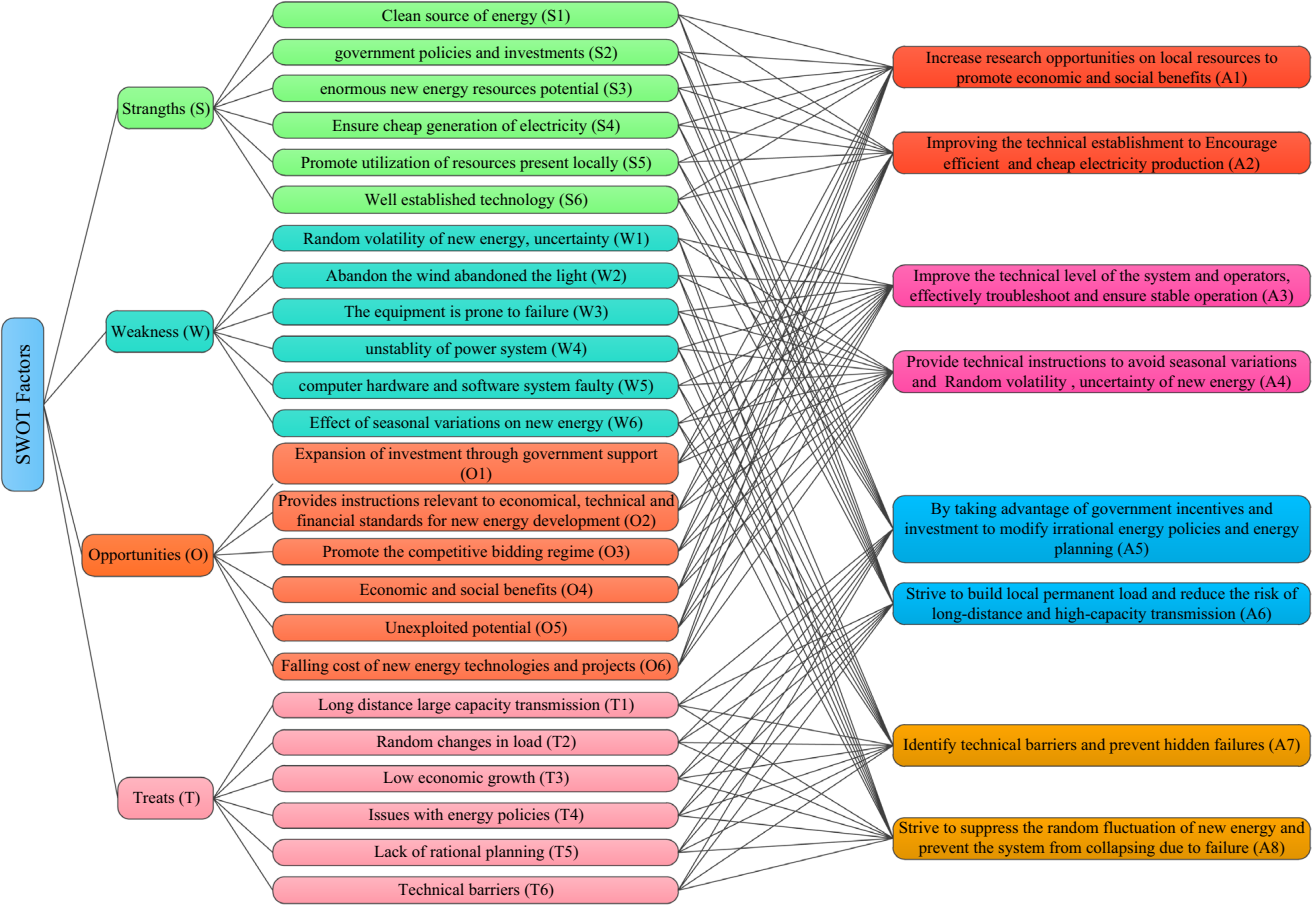
Constructed hierarchical model of SWOT matrix.

The SWOT factors are determined using the collected expert opinions, related studies and industry reports. These factors are listed in Fig. 4.

### Case study

After gathering the SWOT factors, organizations usually use the AHP method to quantify the priority order of strategies and calculate the weights of the factors.

The SWOT matrix and its hierarchical structure constructed by 24 factors and 8 improvement strategies are shown in Fig. 4. A group of experts determine the priorities of SWOT dimensions and factors based on Table 1. The calculated weight values calculated and the results are given in Tables 3, 4, 5, 6, and 7. (C.R. ≤ 0.1).

**Table 3:** Evaluation of SWOT dimensions.

Dimensions	Matrix in linguistic terms				Matrix in fuzzy terms				Weights
	S	W	O	T	S	W	O	T	
S	1	AS	PS	MS	1	[6, 8]	[1, 2]	[2, 4]	0.4284
W		1			[1/8,1/6]	1	[1/6,1/4]	[1/4,1/2]	0.0559
O		SS	1	MS	[1/2,1]	[4, 6]	1	[2, 4]	0.3732
T		MS		1	[1/4,1/2]	[2, 4]	[1/4,1/2]	1	0.1425

(C.R. = 0.016).

**Table 4:** Evaluation criteria of the Strengths.

Strengths	Matrix in linguistic terms						Matrix in fuzzy terms						Local weights
	S1	S2	S3	S4	S5	S6	S1	S2	S3	S4	S5	S6	
S1	1	MS		AS	MS		1	[2, 4]	[1/8,1/6]	[6, 8]	[2, 4]	[1/4,1/2]	0.1495
S2		1		MS	SS	PS	[1/4,1/2]	1	[1/6,1/4]	[2, 4]	[4, 6]	[1, 2]	0.1622
S3	AS	SS	1	AMS	SS		[6, 8]	[4, 6]	1	[8, 10]	[4, 6]	[1/6,1/4]	0.2077
S4				1		SS	[1/8,1/6]	[1/4,1/2]	[1/10,1/8]	1	[1/4,1/2]	[4, 6]	0.1266
S5				MS	1		[1/4,1/2]	[1/6,1/4]	[1/6,1/4]	[2, 4]	1	[1/4,1/2]	0.1761
S6	MS		SS		MS	1	[2, 4]	[1/2,1]	[4, 6]	[1/6,1/4]	[2, 4]	1	0.1779

(C.R. = 0.098).

**Table 5:** Evaluation criteria of the Weaknesses.

Weaknesses	Matrix in linguistic terms						Matrix in fuzzy terms						Local weights
	W1	W2	W3	W4	W5	W6	W1	W2	W3	W4	W5	W6	
W1	1	AS			SS	PS	1	[6, 8]	[1/4,1/2]	[1/4,1/2]	[4, 6]	[1, 2]	0.1880
W2		1		MS	PS		[1/8,1/6]	1	[1/4,1/2]	[2, 4]	[1, 2]	[1/2,1]	0.1419
W3	MS	MS	1	PS	PS	MS	[2, 4]	[2, 4]	1	[1, 2]	[1, 2]	[2, 4]	0.2359
W4	MS			1			[2, 4]	[1/4,1/2]	[1/2,1]	1	[1/4,1/2]	[1/4,1/2]	0.1402
W5				MS	1	PS	[1/6,1/4]	[1/2,1]	[1/2,1]	[2, 4]	1	[1, 2]	0.1322
W6		PS		MS		1	[1/2,1]	[1, 2]	[1/4,1/2]	[2, 4]	[1/2,1]	1	0.1617

(C.R. = 0.052).

**Table 6:** Evaluation criteria of the opportunities.

Opportunities	Matrix in linguistic terms						Matrix in fuzzy terms						Local weights
	O1	O2	O3	O4	O5	O6	O1	O2	O3	O4	O5	O6	
O1	1	PS		SS			1	[1, 2]	[1/4,1/2]	[4, 6]	[1/4,1/2]	[1/6,1/4]	0.1919
O2		1	PS	MS	MS	PS	[1/2,1]	1	[1, 2]	[2, 4]	[2, 4]	[1, 2]	0.2033
O3	MS		1			PS	[2, 4]	[1/2,1]	1	[1/4,1/2]	[1/6,1/4]	[1, 2]	0.1438
O4			MS	1		PS	[1/6,1/4]	[1/4,1/2]	[2, 4]	1	[1/6,1/4]	[4, 6]	0.1584
O5	MS		SS	SS	1		[2, 4]	[1/4,1/2]	[4, 6]	[4, 6]	1	[1/4,1/2]	0.1308
O6	SS				MS	1	[4, 6]	[1/2,1]	[1/2,1]	[1/6,1/4]	[2, 4]	1	0.1719

(C.R. = 0.093).

**Table 7:** Evaluation criteria of the Threats.

Threats	Matrix in linguistic terms						Matrix in fuzzy terms						Local weights
	T1	T2	T3	T4	T5	T6	T1	T2	T3	T4	T5	T6	
T1	1		MS	AS	SS		1	[1/2,1]	[2, 4]	[6, 8]	[4, 6]	[1/2,1]	0.2096
T2	PS	1		SS	MS	MS	[1, 2]	1	[1/2,1]	[4, 6]	[2, 4]	[2, 4]	0.1961
T3		PS	1	MS		SS	[1/4,1/2]	[1, 2]	1	[2, 4]	[1/2,1]	[4, 6]	0.1503
T4				1			[1/8,1/6]	[1/6,1/4]	[1/4,1/2]	1	[1/2,1]	[1/2,1]	0.1389
T5			PS	PS	1	MS	[1/6,1/4]	[1/4,1/2]	[1, 2]	[1, 2]	1	[2, 4]	0.1569
T6	PS			PS		1	[1, 2]	[1/4,1/2]	[1/6,1/4]	[1, 2]	[1/4,1/2]	1	0.1481

(C.R. = 0.077).

**Figure 5:**
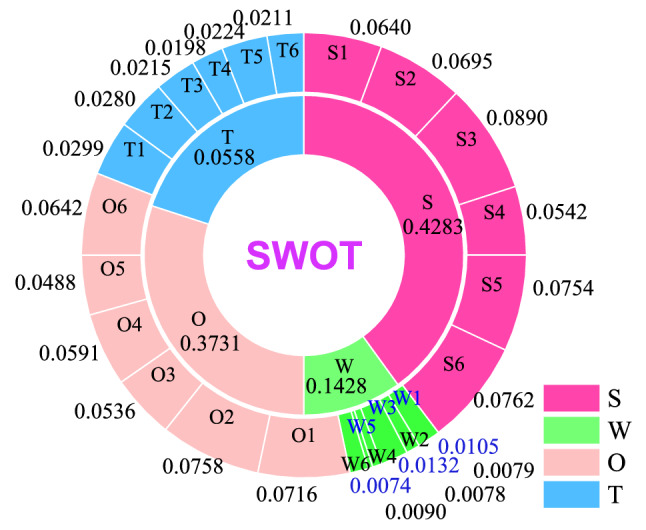
Weight distribution of the SWOT dimensions and factors.

Furthermore, Fig. 5 presents to show the distribution of the weights of SWOT dimensions and factors based on their global weights.

The ranked order of SWOT is obtained as S > O > T > W. Through the strengths of HM power grid, seizing more opportunities and daring to threats will effectively overcome the weaknesses and make the online education more successful. Here, the ranked order of the strength factors is obtained as S3 > S6 > S5 > S2 > S1 > S4. Firstly, vigorously develop the rich potential of new energy resources in HM power grid and develop the local new energy resources through advanced technology. Secondly, through the support of government policies and investment, reduce the cost of power production and benefit the public. Furthermore, the ranked order of the weakness factors is obtained as W3 > W1 > W6 > W2 > W4 > W5. Due to long service time of power grid equipment, coupled with the strong uncertainty of random fluctuation of new energy itself and the impact of quarterly changes on new energy, the system is prone to collapse and various faults, thus affecting the normal operation and power generation of the power system, which corresponds to an inevitable weakness of the HM power grid. Progressively, the ranked order of the opportunity factors is obtained as O2 > O1 > O6 > O4 > O3 > O5. By expanding the government policies, financial support and bidding opportunities, we will enable economic and technical support for the development of new energy, vigorously develop the undeveloped new energy, reduce the power production cost of new energy, and bring the economic and social benefits to the society. Lastly, the ranked order of the threat factors is extracted as T1 > T2 > T5 > T3 > T6 > T4. For North China power grids, especially the HM power grid, large-scale long-distance transmission, “West to East power transmission”, is not only a great project, but also a great technical challenge. The strong randomness of load and the lack of appropriate power transmission planning leads to power transmission security problems. At the same time, such power transmission requires strong support from economy, technology and government policies.

The alternative strategies shown in Fig. 4 are evaluated using the MARCOS method over the SWOT factors by the experts using linguistic terms given in Table 2, to obtain a group decision matrix given in Table 8.

**Table 8:** Evaluation of alternatives by the group of experts.

Strategies	S1	S2	S3	S4	S5	S6	W1	W2	W3	W4	W5	W6	O1	O2	O3	O4	O5	O6	T1	T2	T3	T4	T5	T6
*A*_1_	MG	MG	RG	P	M	MP	HP	RP	P	MP	M	HG	G	RG	M	RG	G	M	MG	MP	M	M	M	M
*A*_2_	M	MG	MG	RG	RG	M	HP	HP	P	MP	G	M	HG	RG	G	HG	MG	RG	M	MP	MG	M	M	MP
*A*_3_	P	M	M	HP	HG	G	MG	MG	G	HG	HG	M	P	HP	G	HP	RP	M	G	G	P	MP	M	HG
*A*_4_	P	M	MP	P	P	RG	RG	HG	M	M	G	RG	MP	M	RG	P	HP	HP	M	G	MG	RG	RG	HG
*A*_5_	G	RG	G	G	G	MG	HP	HP	G	G	MG	M	HG	RG	MG	HG	M	HP	HG	RG	HG	G	M	HG
*A*_6_	G	MG	HG	RG	RG	HG	P	P	MG	MG	M	RG	RG	M	MG	G	HG	MG	RG	RG	G	MG	RG	HG
*A*_7_	G	M	RG	MP	P	HP	G	G	RG	RG	RG	HG	P	M	MP	HP	HP	M	RG	RG	M	G	G	RG
*A*_8_	RG	P	RG	HP	G	G	RG	RG	RP	HP	HP	RG	P	M	HP	MP	M	MP	RG	RG	G	MP	MP	HG

**Figure 6:**
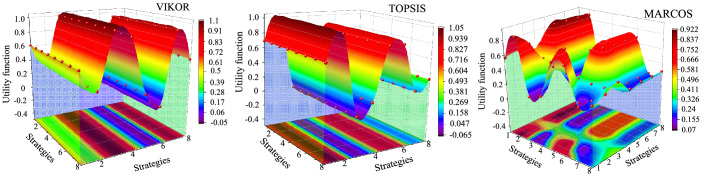
Three dimensional distribution of utility function values of three methods.

The extended initial fuzzy matrix ${\tilde{\mathbf{X}}}$ of MARCOS method is first created, including fuzzy anti-ideal ${\tilde{\mathbf{A}}}\left( {AAI} \right)$ and ideal ${\tilde{\mathbf{A}}}\left( {AI} \right)$ solutions. Extended initial fuzzy matrix is then normalized based on the benefit criteria of the proposed SWOT model in step 7. Here, all SWOT factors are benefit criteria. The weighted fuzzy matrix ${\tilde{\mathbf{V}}}$ created using global weights of the SWOT factors is given in Fig. 6. The utility degree ${\tilde{\mathbf{K}}}_{j}$, ${\tilde{\mathbf{A}}}_{j}$ values, utility functions for the ideal $f(K_{j}^{ + } )$ and anti-ideal $f(K_{j}^{ - } )$ solutions, and $\tilde{T}_{j}$ values of alternative strategies are calculated and provided in Table 9. In addition, the defuzzification of ${\tilde{\mathbf{K}}}_{j}^{ - }$, ${\tilde{\mathbf{K}}}_{j}^{ + }$, $f(K_{j}^{ + } )$, $f(K_{j}^{ - } )$ values and utility functions $f(K_{j} )$ of alternative strategies are obtained and the results are given in Table 10. The ranked order of the strategies is obtained as *A*_2_ > *A*_6_ > *A*_5_ > *A*_1_ > *A*_8_ > *A*_7_ > *A*_4_ > *A*_3_.

**Table 9:** Obtained ${\tilde{\mathbf{A}}}_{j}$, ***K̃***_*j*_ (${\tilde{\mathbf{K}}}_{j}^{ - }$,${\tilde{\mathbf{K}}}_{j}^{ + }$) and ${\tilde{\mathbf{T}}}_{j}$ values.

${\tilde{\mathbf{A}}}_{j}$	$(A_{j}^{l} ,A_{j}^{m} ,A_{j}^{u} )$			${\tilde{\mathbf{K}}}_{j}^{ - }$	$(K_{j}^{ - l} ,K_{j}^{ - m} ,K_{j}^{ - u} )$			${\tilde{\mathbf{K}}}_{j}^{ + }$	$(K_{j}^{ + l} ,K_{j}^{ + m} ,K_{j}^{ + u} )$			${\tilde{\mathbf{T}}}_{j}$	$(t_{j}^{l} ,t_{j}^{m} ,t_{j}^{u} )$		
$\tilde{A}_{aj}$	(3.0559	2.9057	1.4752)	${\tilde{\mathbf{K}}}_{aj}^{ - }$	(1.0043	0.9760	1.0417)	${\tilde{\mathbf{K}}}_{aj}^{ + }$	(3.0698	2.8365	1.5369)	${\tilde{\mathbf{T}}}_{aj}$	(4.0742	3.8125	2.5786)
$\tilde{A}_{1}$	(2.2986	2.3686	1.3232)	$\tilde{K}_{1}^{ - }$	(0.7522	0.8151	0.8970)	$\tilde{K}_{1}^{ + }$	(2.2990	2.3690	1.3234)	$\tilde{t}_{1}$	(3.0512	3.1842	2.2204)
$\tilde{A}_{2}$	(3.0692	2.8360	1.5351)	$\tilde{K}_{2}^{ - }$	(1.0043	0.9760	1.0407)	$\tilde{K}_{2}^{ + }$	(3.0698	2.8365	1.5354)	$\tilde{t}_{2}$	(4.0742	3.8125	2.5761)
$\tilde{A}_{3}$	(1.0265	1.0748	0.8931)	$\tilde{K}_{3}^{ - }$	(0.3359	0.3699	0.6054)	$\tilde{K}_{3}^{ + }$	(1.0267	1.0750	0.8932)	$\tilde{t}_{3}$	(1.3626	1.4449	1.4986)
$\tilde{A}_{4}$	(1.2677	1.3590	0.9783)	$\tilde{K}_{4}^{ - }$	(0.4148	0.4677	0.6632)	$\tilde{K}_{4}^{ + }$	(1.2680	1.3593	0.9785)	$\tilde{t}_{4}$	(1.6828	1.8270	1.6416)
$\tilde{A}_{5}$	(2.8222	2.3737	1.5299)	$\tilde{K}_{5}^{ - }$	(0.9235	0.8169	1.0371)	$\tilde{K}_{5}^{ + }$	(2.8228	2.3741	1.5302)	$\tilde{t}_{5}$	(3.7463	3.1910	2.5673)
$\tilde{A}_{6}$	(2.6970	2.4538	1.5366)	$\tilde{K}_{6}^{ - }$	(0.8826	0.8445	1.0417)	$\tilde{K}_{6}^{ + }$	(2.6976	2.4543	1.5369)	$\tilde{t}_{6}$	(3.5801	3.2987	2.5786)
$\tilde{A}_{7}$	(1.3310	1.5032	1.0056)	$\tilde{K}_{7}^{ - }$	(0.4355	0.5173	0.6817)	$\tilde{K}_{7}^{ + }$	(1.3312	1.5035	1.0058)	$\tilde{t}_{7}$	(1.7668	2.0208	1.6874)
$\tilde{A}_{8}$	(1.5342	1.7498	1.1026)	$\tilde{K}_{8}^{ - }$	(0.5021	0.6022	0.7474)	$\tilde{K}_{8}^{ + }$	(1.5346	1.7501	1.1028)	$\tilde{t}_{8}$	(2.0366	2.3523	1.8502)
$\tilde{A}_{jd}$	(0.9998	0.9998	0.9998)	${\tilde{\mathbf{K}}}_{jd}^{ - }$	(0.3359	0.3699	0.6054)	${\tilde{\mathbf{K}}}_{jd}^{ + }$	(1.0267	1.0750	0.8932)	${\tilde{\mathbf{T}}}_{jd}$	(1.3626	1.4449	1.4986)

**Table 10:** Results of fuzzy MARCOS method and ranked order of the strategies.

Strategies	${\tilde{\mathbf{K}}}_{j}^{ - }$	${\tilde{\mathbf{K}}}_{j}^{ + }$	$f(K_{j}^{ - } )$	$f(K_{j}^{ + } )$	$f(K_{j} )$	Order
*A*_1_	0.8199	2.0901	0.2246	0.5726	0.5597	4
*A*_2_	0.9992	2.5696	0.2737	0.7039	0.8760	1
*A*_3_	0.4203	1.0175	0.1151	0.2787	0.1275	8
*A*_4_	0.5034	1.2413	0.1379	0.3400	0.1898	7
*A*_5_	0.8986	2.2753	0.2462	0.6233	0.6801	3
*A*_6_	0.9033	2.2858	0.2474	0.6262	0.6875	2
*A*_7_	0.5380	1.3360	0.1474	0.3660	0.2200	6
*A*_8_	0.6135	1.5344	0.1681	0.4203	0.2930	5

The results of this study essentially demonstrated that the most important dimensions that the State Grid should focus on are the strengths and opportunities concerning the HM power grid, where the internal and external factors have almost the same importance. Therefore, we strive to maximize our strengths, weaken our weaknesses, dare to threats and seize the opportunities. Moreover, the ranked order of the strategies reveals that the three most important digital transformation strategies are *A*_2_, *A*_6_ and *A*_5_. These three strategies are defined as “improving the technical establishment to encourage efficient and cheap electricity production”, “strive to build local permanent load and reduce the risk of long-distance and high-capacity transmission”, and “taking advantage of government incentives and investment to modify the irrational energy policies and energy planning”, respectively.

### Comparative analysis

This section provides the comparative analysis that is conducted to validate the proposed methodology. The comparison is made against the VIKOR and TOPSIS methods. Computational steps of VIKOR^20^ and TOPSIS^32^ are performed, and the corresponding results of VIKOR and TOPSIS are given in Tables 11 and 12, respectively. Besides, the utility function distribution in a three-dimensional space is shown in Fig. 6.

**Table 11:** Results of fuzzy VIKOR method and the ranked order of the strategies.

Strategies	*S*_*j*_	Order	*R*_*j*_	Order	*Q*_*j*_	Order
*A*_1_	1.4221	5	0.4805	5	0.6110	5
*A*_2_	1.1726	6	0.4153	6	0.3383	3
*A*_3_	2.0917	1	0.7082	1	1.0000	8
*A*_4_	1.9717	2	0.6669	2	0.9101	6
*A*_5_	0.9767	7	0.3514	7	0.1956	2
*A*_6_	0.6742	8	0.2743	8	0.0000	1
*A*_7_	1.7801	3	0.6059	3	0.9351	7
*A*_8_	1.4797	4	0.5067	4	0.5519	4

**Table 12:** Results of fuzzy TOPSIS method and the ranked order of the strategies.

Strategies	$D_{j}^{ + }$	$D_{j}^{ - }$	*C*_*j*_	Order
*A*_1_	0.4794	1.0724	0.6911	4
*A*_2_	0.0000	1.5518	1	1
*A*_3_	1.5518	0.0000	0	8
*A*_4_	1.3281	0.2237	0.1442	7
*A*_5_	0.2942	1.2576	0.8104	3
*A*_6_	0.2838	1.2680	0.8171	2
*A*_7_	1.2333	0.3184	0.2052	6
*A*_8_	1.0350	0.5168	0.3330	5

**Figure 7:**
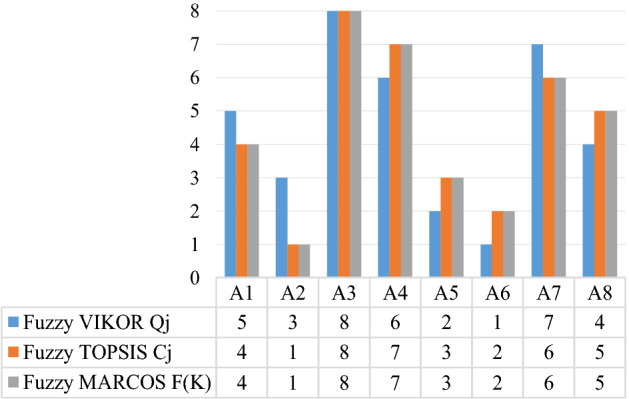
Compared ranked orders of the strategies.

Furthermore, the ranked orders of the alternatives obtained from fuzzy VIKOR, TOPSIS, and MARCOS methods are compared in Fig. 7, which are found as VIKOR—— *A*_6_ > *A*_5_ > *A*_2_ > *A*_8_ > *A*_1_ > *A*_4_ > *A*_7_ > *A*_3_; TOPSIS—— *A*_2_ > *A*_6_ > *A*_5_ > *A*_1_ > *A*_8_ > *A*_7_ > *A*_4_ > *A*_3_. According to the results, the solutions from these three compared methods for the evaluation of operation security strategies produce similar results. There are both distance-based method, which are similar in their approach. However, the MARCOS method is a relatively new and practical method compared to the other two methods. Compared to the outcomes of other prominent MCDM methods, the integrated AHP-MARCOS methodology provides highly consistent final values, validating the potential of proposed method in solving similar MCDM problems.

### Sensitivity analysis

**Figure 8:**
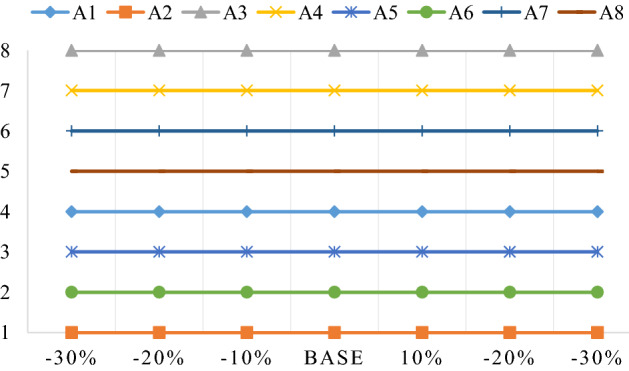
The comparison of the ranked orders of the strategies.

The sensitivity analysis can confirm the final prioritization of changes in priority weights of criteria. Table 13 shows the SWOT dimensions have ± 10%, ± 20%, and ± 30% ranking of global weights change. Following that, through the fuzzy MARCOS method, update the utility function *F*(*K*) values based on these weights. Next, ranked orders of the strategies alternatives are obtained based on the updated utility function values as shown in Fig. 8, which demonstrates the robustness and credibility of the MARCOS method. Furthermore, “improving the technical establishment to encourage efficient and cheap electricity production” is the most appropriate strategy in most cases.

**Table 13:** Weight values of the SWOT dimensions in different cases.

SWOT dimensions	- 30%	- 20%	- 10%	Base	10%	20%	30%
S	− 0.12849	− 0.08566	− 0.04283	0.4283	0.04283	0.08566	0.12849
W	− 0.01674	− 0.01116	− 0.00558	0.0558	0.00558	0.01116	0.01674
O	− 0.11193	− 0.07462	− 0.03731	0.3731	0.03731	0.07462	0.11193
T	− 0.04284	− 0.02856	− 0.01428	0.1428	0.01428	0.02856	0.04284

## Conclusion and perspective

New energy offers enormous potential to generate electricity in Northwest China regions, which could potentially be enough to meet overall China’s energy demands. The vigorous development and utilization of new energy at HM power grid can not only reduce the dependence on traditional energy such as coal, oil and natural gas, but also improve the problem of energy shortage, create new employment opportunities, protect the natural environment, and make the rural regions with significant new energy potential wealthier. However, with the integration of new energy and microclimate-sensitive loads, power systems are becoming increasingly complex and vulnerable to faults. As the main power generation area of Northwest China power grid, the safe and economic operation of HM power grid is becoming a more important and complex problem in the field of power system security operation. The strategic evaluation of this paper shows that the proposed method can be effectively used to determine the best strategies with highest priority, and illustrates the importance of establishing the right strategy to exploit a significant developing opportunity and highlight the strength of rapidly growing new energy power grid.

The major contributions of this paper can be summarized as follows:

- This article is the first study focusing on SWOT-AHP-MARCOS method for new energy power system operation security.
- This study contributes to the HM power grid security operation prospective strategy selection problem by developing a new evaluation model. The effectiveness of the presented approach is validated via comparative analysis and sensitivity analysis. The results as follow:

It is concluded that the three most important strategies are *A*_2_, *A*_6_ and *A*_5_. These are “improving the technical establishment to encourage efficient and cheap electricity production”, “strive to build local permanent load and reduce the risk of long-distance and high-capacity transmission”, and “taking advantage of government incentives and investment to modify the irrational energy policies and energy planning”, respectively. Northwest region is a poor area in China with underdeveloped economy. Although it has a great potential for new energy, this energy cannot be consumed locally. Therefore, the government advocates the policy of “power transmission from the west to the East” to invest in the energy field in the northwest region. First, it aims to solve the problem of energy shortage in the eastern developed regions. Second, it aims to promote the economic development of the northwest region through the advantages of high new energy storage, so as to achieve the “Chinese dream” of energy coexistence and common prosperity. In this regard, the results of the presented SWOT analysis showed that despite various challenges, HM power grid could still provide numerous benefits to the country over conventional non-new energy resources in terms of production cost, environmental aspects with additional prospects of endless new energy potential, which will greatly promote the economic development of Northwest China.

In the future, it is worthwhile to further study the following aspects:

- The MCDM methods such as HFL-AHP, ANP, CM, CRITIC, DEA, ELECTRE, MABAC, TOPSIS, and VIKOR can be applied to the similar-type of decision-problem.
- Based on the construction of SWOT index system, the method in this paper can be used to evaluate the power system development prospect among different regions in the world.

However, one of the limitations of this study is the identification of the items within the SWOT factors. During the process, the researchers’ subjectivity may have affected the results. We believe that if the items were derived directly through interviews with experts and the top four items among them were selected, we could secure more objective results.

## Supplementary Information

Supplementary Information 1.

Supplementary Information 2.

## Data Availability

The datasets used and/or analysed during the current study available from the corresponding author on reasonable request.
